# RXR Expression in Marine Gastropods with Different Sensitivity to Imposex Development

**DOI:** 10.1038/s41598-020-66402-1

**Published:** 2020-06-11

**Authors:** Sebastián Giulianelli, Mónica A. Primost, Claudia Lanari, Gregorio Bigatti

**Affiliations:** 1 Instituto de Biología de Organismos Marinos, IBIOMAR-CCT CENPAT-CONICET, Puerto Madryn, Argentina; 2 Instituto de Biología y Medicina Experimental, IBYME-CONICET, Buenos Aires, Argentina; 3 Grupo de Investigación y Desarrollo Tecnológico en Acuicultura y Pesca (GIDTAP), Universidad Tecnológica Nacional. Facultad Regional Chubut (UTN-FRCh), Puerto Madryn, Argentina; 4 Universidad Nacional de la Patagonia San Juan Bosco, Puerto Madryn, Argentina; 5 Universidad Espíritu Santo, Guayaquil, Ecuador

**Keywords:** Proteins, Marine biology, Animal physiology

## Abstract

The superposition of male sexual characteristics in female marine gastropods (imposex) represents one of the clearest ecological examples of organotin-mediated endocrine disruption. Recent evidences suggest that signaling pathways mediated by members of the nuclear receptor superfamily, RXR and PPARγ, are involved in the development of this pseudohermaphroditic condition. Here, we identified significant differences in RXR expression in two caenogastropod species from Nuevo Gulf, Argentina, *Buccinanops globulosus* and *Trophon geversianus*, which present clear contrast in imposex incidence. In addition, *B. globulosus* males from a polluted and an unpolluted area showed differences in RXR expression. Conversely, PPARγ levels were similar between both analyzed species. These findings indicate specie-specific RXR and PPARγ expression, suggesting a major role of RXR in the induction of imposex.

## Introduction

Endocrine disrupting chemicals are compounds that alter the normal functioning of the endocrine system of both humans and wildlife^1^. One of most relevant examples of environmental endocrine disruption in marine gastropods is the phenomenon of imposex^2^, described as an irreversible syndrome in which a female develops male-type genital organs, such as the penis and vas deferens^3,4^. Imposex is intimately associated with tributyltin (TBT) marine environmental pollution^5^. TBT is a common compound in marine antifouling paints, currently used in several countries^6^ despite having been banned worldwide for more than 10 years^7^. Although imposex levels appear to be reduced in some regions of the planet^8,9^ after global ban of organotin antifoulant paints, assessing the incidence of imposex as a biomarker of TBT contamination seems to be a valuable indicator in other regions of the world such as Latin America^10–12^ or South Africa^13^ where TBT-based antifouling paints are still in use.

The use of TBT, under controlled laboratory conditions, diluted in the water or directly injected to gastropods, was able to induce imposex in several caenogastropods^14–19^.

Different mechanisms have been proposed to explain the induction of imposex by TBT such as an increase in androgen levels, the involvement of penis morphogenetic/retrogressive factor, or even an increase in the neuropeptide alanine-proline-glycine-tryptophan amide^20^. However, a considerable body of evidence indicates that TBT-induced imposex involves the abnormal modulation of the retinoid X receptor (RXR) signaling pathway^20–23^. RXR are members of the nuclear receptor superfamily of transcription factors^24^. They are activated by retinoic acid, the main derivative of vitamin A, involved in multiple signaling pathways critical for embryonic development, metabolic processes, differentiation and apoptosis in vertebrates^25,26^. Although retinoid physiology is poorly understood in marine gastropods^27^, recent findings support their role during gonad maturation^28^. RXR was cloned in different caenogastropods, namely, *Thais clavigera*^18^, *Nucella lapillus*^14^, *Ilyanassa obsoleta*^29^, *Hexaplex trunculus*^15^, *Plicopurpura pansa*^30^ and *Babylonia japonica*^31^. Interestingly, the DNA and ligand binding domains (DBD and LBD respectively) had almost 100% and 90–80% identities, respectively, between gastropod and mouse/human RXRs^32^.

It has been shown that TBT binds RXR and activates the RXR-peroxisome proliferator-activated receptor gamma (PPARγ) heterodimer^33^. Direct administration of 9-cis retinoic acid (9cRA) induced imposex in *T. clavigera*^18,34^ and *N. lapillus*^14^. However, the application of rosiglitazone (Rosi), a PPARγ ligand, to *N. lapillus*, induced imposex in the absence of TBT^35^. All these data suggests the direct association between TBT-RXR-PPARγ with imposex development.

The nassarid gastropod *Buccinanops globulosus* (Kiener, 1834) inhabits Nuevo Gulf and is distributed along the Argentinean coasts in sandy bottoms of shallow waters^36^. The genus *Buccinanops* has been affected by imposex in Argentina^37,38^. *Trophon geversianus* (Pallas, 1774) is a muricid gastropod widely distributed along the Southern Atlantic and Pacific coasts^39–41^. The specie is present in subtidal and intertidal habitats from Nuevo Gulf. Imposex incidence is very low in *T. geversianus*, even in harbor areas with TBT contamination. In contrast, *B. globulosus* presents high sensitivity to imposex incidence even at low environmental TBT concentrations^41^.

Working with species that inhabit the same area being exposed to the same environmental conditions but show physiological differences in the development of imposex, will allow us to expand our knowledge on the metabolic pathways altered by organotin compounds. Thus, the specific aim of this study was to evaluate the expression of RXR and PPARγ in two caenogastropods from Nuevo Gulf, Argentina, *B. globulosus* and *T. geversianus*, to explain some aspects of its differential sensibility to imposex development.

## Materials and Methods

### Collection of animals

Sexually mature males and females of *B. globulosus* and *T. geversianus* were collected during July-August 2016 from two places in Nuevo Gulf, with the same physical conditions and separately by 20 km, Cerro Avanzado beach (CA), a zone with scarce maritime traffic and very low imposex incidence, and from Luis Piedra Buena Harbor (LPBH), located in the city of Puerto Madryn^42^ (Supplementary Fig. S1). LPBH is an area with high maritime traffic characterized by the presence of commercial, fishing and recreational vessels. A total of 895 boats with up to 294 m length, arrived in the city in 2016/2017 season^43^. Aluminum derivates, porfids and others materials are transported during the year. High levels of trace metals, polycyclic aromatic hydrocarbons (PAHs) and TBT were previously recorded in sediments and organisms^41,44–48^.

### Tissue preparation

After removal of the shell, the percentage of imposex-affected females was calculated in terms of penis and vas deferent presence, and the relative penis length index (RPLI) was calculated^49^. Male penis and penis/penis-forming area from imposex-affected female were excised out and fixed in formalin solution (4% in PBS) or immediately frozen at −80 °C.

### Immunohistochemistry (IHC)

Sample sections of formalin-fixed, paraffin-embedded tissues were stained with hematoxylin-eosin (H&E) or reacted with RXR (sc-774) and PPARγ (sc-7196) antibodies (Santa Cruz Biotechnology) using the avidin-biotin peroxidase complex technique (Vectastain Elite ABC kit; Vector Laboratories). Briefly, 3 penis sections from five independent individuals of both species were rehydrated from xylene to 70% ethanol passing through decreased graded ethanol, before endogenous peroxidase activity inhibition (10% H_2_O_2_ in 70% ethanol). Antigen retrieval with HCl 2 N was performed before immunostaining. After PBS washes, sections were blocked (2.5% BSA in PBS) and incubated overnight at 4 °C with the primary antibody. After biotin-conjugated secondary antibody (incubation for 1 h at room temperature, the reaction was developed using the DAKO Liquid DAB + Substrate Chromogen System (K3468, DAKO) according to the manufacturer’s protocol under microscopic control. Specimens were counterstained with hematoxylin, dehydrated and mounted. Positive cells were counted in 10 high-power fields (HPFs) of each section, using 1000× magnification, and expressed as the mean ± SEM of the percentage of the ratios between the number of events and the cell number/HPF.

### Tissue extracts and western blot

Frozen tissues from five independent individuals of both species were homogenized in ice-cold TEDGS 10% buffer (50 mM Tris pH = 7.4, 7.5 mM EDTA, 0.5 mM dithiothreitol, 10% glycerol, 0.25 M sucrose), including protease inhibitors. The homogenate was centrifuged (20 min, 12000 rpm at 4 °C) and total protein concentration in each supernatant was determined by Lowry method^50^. Equivalent amounts of protein (100 μg) from tissue lysates were separated on discontinuous polyacrylamide gels and detected by western blot. Membranes were probed with RXR (sc-774), PPARγ (sc-7273) or β-Actin (sc-47778) antibodies (Santa Cruz Biotechnology) overnight at 4 °C, and then incubated with horse anti-mouse or goat anti-rabbit peroxidase-conjugated secondary antibody (Vector Laboratories). The luminescent signal was generated by enhanced chemiluminescence (ECL) method and the blots were exposed to a medical X-ray film (ortho CP-GU, AGFA). Band intensity was determined by densitometry using ImageJ 1.47 v software (https://imagej.nih.gov/ij/download.html)^51^.

### Statistical analysis

ANOVA and the Tukey multiple post *t* test were used to analyze the differences of means of multiple samples; the Student’s *t* test was used to compare the means of two different groups. In all graphs, the mean ± SEM is shown, and experiments were repeated at least three times. Significant differences between groups are indicated with asterisks (*p < 0.05; **p < 0.01; ***p < 0.001).

## Results

### Imposex incidence in *B. globulosus* and *T. geversianus*

**Figure 1:**
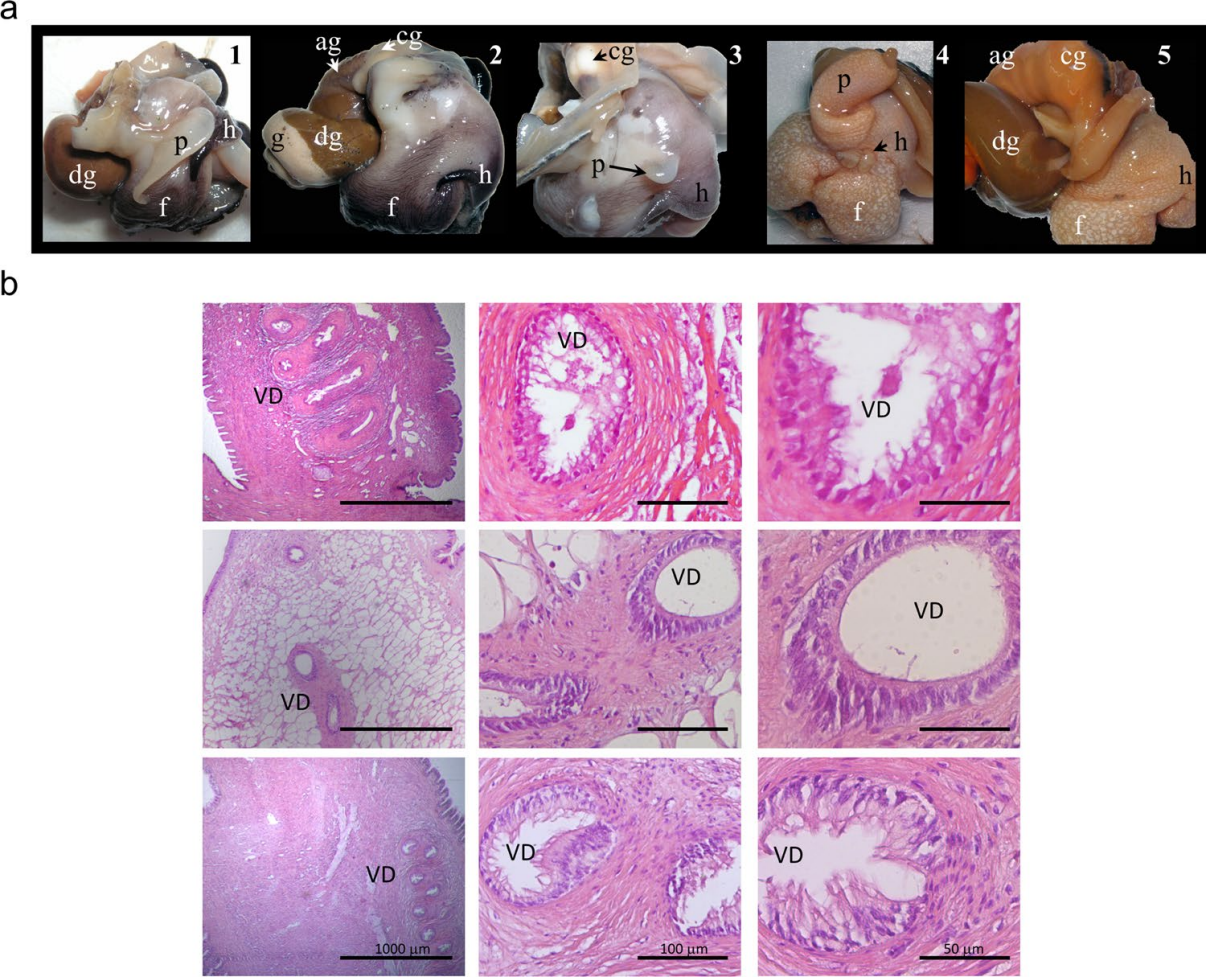
Morphology and histology of *Buccinanops globulosus* and *Trophon geversianus*. (**a**) *B. globulosus* male (1) and normal female (2) from CA and imposex-affected female (3) from LPBH. *T. geversianus* male (4) and female (5) from LPBH. p: penis, dg: digestive gland, ag: albumen gland, cg: capsule gland, h: head, f: foot. (**b**) H&E stains from paraffin sections of penis from male (*top*) and imposex-affected female (*middle*) of *B. globulosus*, and from male of *T. geversianus* (*bottom*). VD: vas deferens.

*B. globulosus* females collected from Luis Piedra Buena Harbor (LPBH) presented 100% imposex incidence with different degrees of penis development (between 0.5 to 7.7 mm of length); meanwhile the opposite was observed for *T. geversianus* specimens (Table 1 and Fig. 1a). No imposex incidence was registered in female gastropods of both species collected in Cerro Avanzado beach (CA).

**Table 1:** Shell and penis length (mean ± SD), together with imposex incidence and RPLI values in *Buccinanops globulosus* and *Trophon geversianus*.

	Site		n	Shell length (mm)	% Imposex	Penis length (mm)	RPLI
*Buccinanops globulosus*	CA	Female	32	38.38 ± 4.11	0	—	0
		Male	8	30.63 ± 2.82	—	14.81 ± 2.79	
	LPBH	Female	26	42.81 ± 5.3	100	3.77 ± 1.51	24.7
		Male	8	30.75 ± 1.75	—	15.26 ± 4.05	
*Trophon geversianus*	CA	Female	27	26.59 ± 1.97	0	—	0
		Male	9	23.33 ± 1.32	—	8.76 ± 1.23	
	LPBH	Female	12	33 ± 3.49	0	—	0
		Male	14	32.93 ± 3.89	—	10.88 ± 1.64

### Histological visualization of imposex

The penis and penis-like structures developed behind the right tentacle in normal male of *B. globulosus* and *T. geversianus* and in imposex-affected females of *B. globulosus* collected from LPBH were histologically examined after H&E staining (Fig. 1b). No morphological differences were observed between penis from male and penis-like structure from imposex-affected females of *B. globulosus*. An internal vas deferens (VD) was observed in the central area of the penis/penis-like structure. The epithelium of the VD was surrounded by smooth muscle layers. The epidermis of the penis/penis-like structure consisted of epidermal cells with mucous cells (Fig. 1b).

### Evaluation of RXR and PPARγ expression

**Figure 2:**
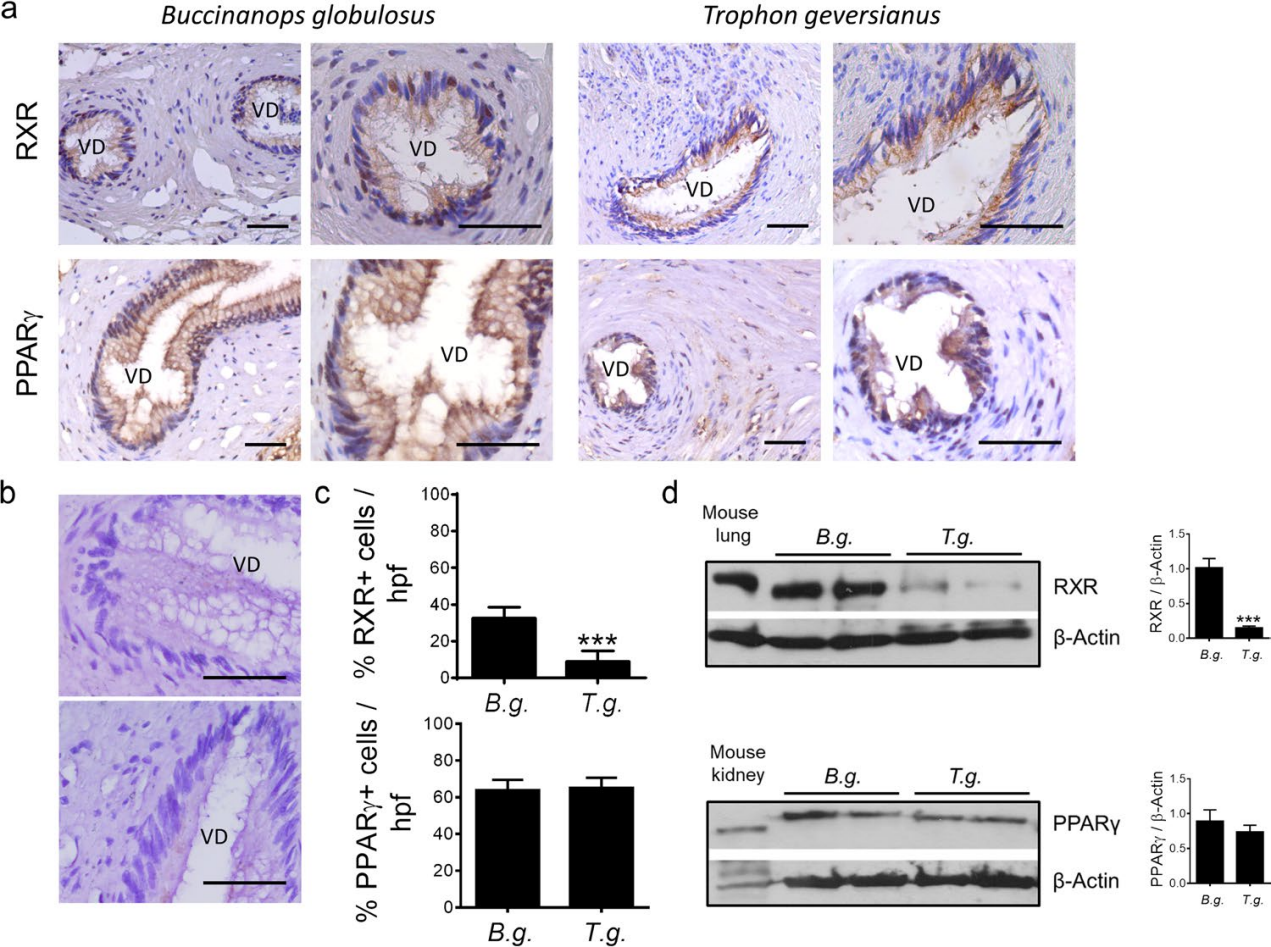
RXR and PPARγ expression in males of *Buccinanops globulosus* and *Trophon geversianus*. (**a**) Immunohistochemical staining for RXR (*top*) and PPARγ (*bottom*) in sections of paraffin-embedded penis of *Buccinanops globulosus* and *Trophon geversianus* males collected in CA.VD: vas deferens. Bar: 50 µm. (**b**) Control assays from (**a**) in which no primary antibody was added. VD: vas deferens. Bar: 50 µm. (**c**) Quantification of positive cells/HPF from experiments depicted in (**a**). ***p < 0.001. *B.g*.: *Buccinanops globulosus*, *T.g*.: *Trophon geversianus*. (**d**) Representative western blots showing RXR (*top left*) and PPARγ (*bottom left*) expression in total extracts from penis of *Buccinanops globulosus* (*B.g*.) and *Trophon geversianus* (*T.g*.) males collected in CA. β-Actin was used as a loading control. The band intensity ratios of RXR and PPARγ expression relative to β–Actin were plotted (*right*, ***p < 0.001). Mouse lung and kidney tissues were used as positive controls. Full-length blots are presented in Supplementary Fig. S3.

Immunohistochemical stains against RXR and PPARγ illustrate the localization of both proteins in the penis of *B. globulosus* and *T. geversianus* male gastropods collected from CA beach (Fig. 2a). RXR and PPARγ were detected in the nuclei of epithelial cells and smooth muscle cells surrounding the epithelium of vas deferens. No immunoreactivity was detected in the absence of primary antibodies (Fig. 2b), confirming binding specificity.

We found a significant lower expression of RXR in penis of *T. geversianus* males in comparison with the same tissue of *B. globulosus* (a similar trend was found in epidermal cells of male penis, and in female gonads and digestive glands, Supplementary Fig. S2). In the case of PPARγ, no differences were observed between both analyzed species (Fig. 2c). These results were validated by western blot (Fig. 2d).

**Figure 3:**
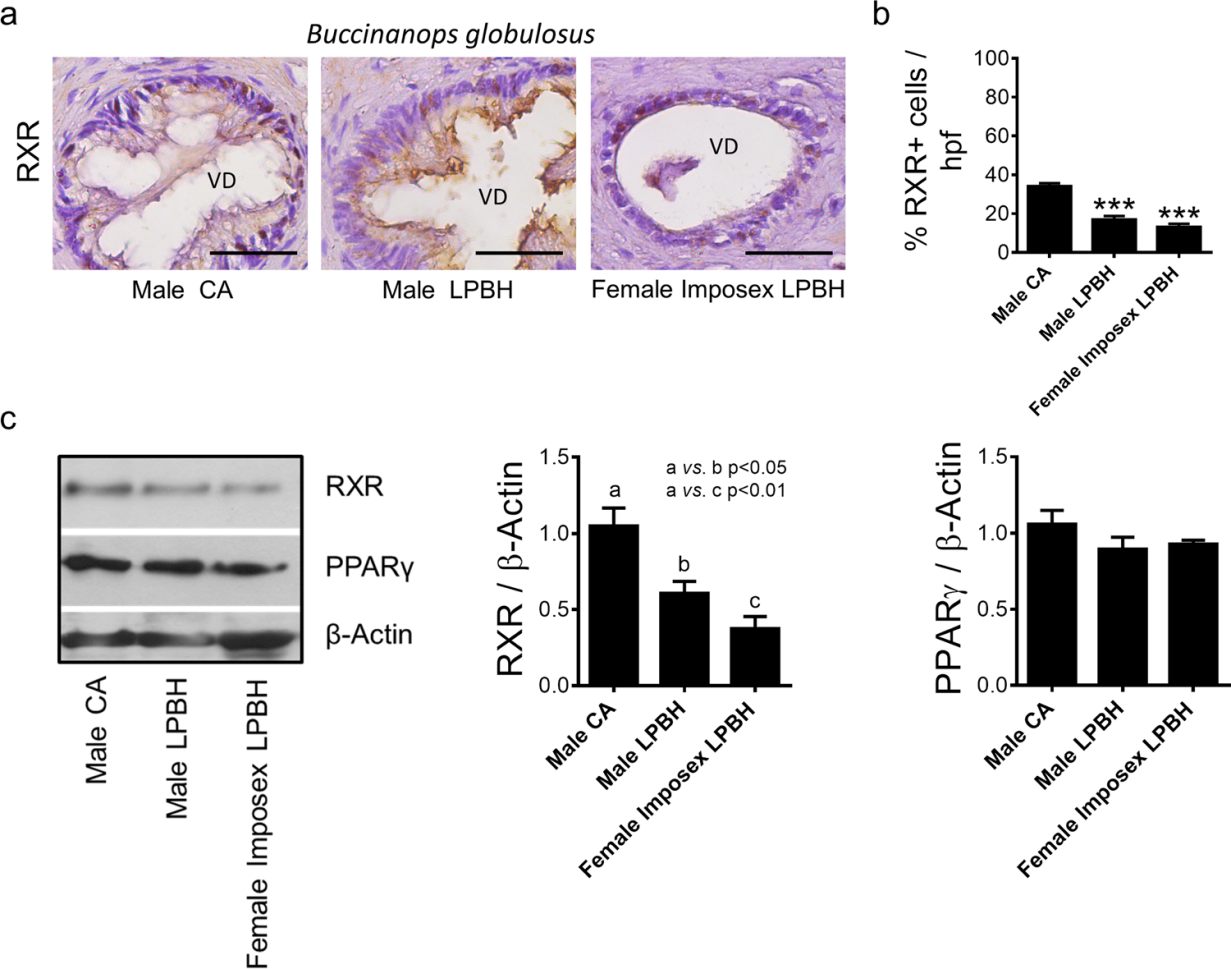
RXR and PPARγ expression in imposex-affected females. (**a**) Immunohistochemical staining for RXR in sections of paraffin-embedded penis of *Buccinanops globulosus* males and imposex-affected females collected in CA and LPBH.VD: vas deferens. Bar: 50 µm. (**b**) Quantification of RXR positive cells/HPF from experiments depicted in (**a**). ***p < 0.001. (**c**) Representative western blots showing RXR and PPARγ (*left*) expression in total extracts from penis of *Buccinanops globulosus* males and imposex-affected females collected in CA and LPBH. β-Actin was used as a loading control. The band intensity ratios of RXR and PPARγ expression relative to β–Actin were plotted (*middle* and *right* respectively). Full-length blots are presented in Supplementary Fig. S4.

To further evaluate the expression of RXR and PPARγ in gastropods from a polluted (LPBH) or unpolluted (CA beach) areas, we used penis tissues from imposex-affected females and males of *B. globulosus*. We observed RXR and PPARγ expression in males from LPBH and CA beach and in imposex-affected *B. globulosus* females collected at LPBH (Fig. 3). Significant differences in RXR penis tissues expression were observed among males from CA and LPBH, and males from CA and imposex-affected females from LPBH, while no differences in PPARγ expression was detected (Fig. 3b,c).

## Discussion

In this study, we compared for the first time the expression of RXR and PPARγ in two caenogastropod species from South Atlantic, which present clear contrasts in imposex incidence. Most of biochemical or molecular studies related to imposex have been performed under experimental laboratory conditions. Ours, is an entirely field work reflecting what occurs in natural conditions underscoring the relevance of the data. We found that penis of *B. globulosus* males express higher levels of RXR than the same tissue of *T. geversianus* males, collected in an unpolluted area (CA) of Nuevo Gulf. These results suggest specie-specific differences in RXR expression that could explain differences registered in penis length between both species (and probably in other neogastropods), and may also be related to their different biological response to environmental contaminants. As was previously mentioned, the hypothesis about the abnormal modulation of RXR as a mechanism by which organotins induce imposex in caenogastropods would turn out to be the most accepted one^20,52,53^. Therefore, RXR could be a central factor in the molecular mechanism involved in differentiation, proliferation, and morphogenesis of penis in male and imposex-affected female gastropods. Nevertheless, the low expression of RXR in penis from *T. geversianus* males, relative to that in *B. globulosus* males, is not an impediment to the normal development of secondary sexual characters as the penis. In addition to the local action that RXR would have in penis development, which would be under the control of the central nervous system (CNS) of gastropods, CNS was described as a tissue with high RXR expression able of being retinoid target^21^. It was proposed that a “penis morphogenetic factor (PMF)” released by CNS under the control of RXR signaling is induced in males during penis differentiation^21^. That may explain the normal development of *T. geversianus* penis even having low levels of RXR expression, relative to *B. globulosus* males. However, this hypothesis and the identification of a PMF in *T. geversianus* and other gastropods need to be addressed. Future proteomic strategies, comparing both species under study, will shed light on further important factors or signaling pathways involved in differentiation, growth and penis formation in males.

Interestingly, no differences were observed in PPARγ expression between the species studied, highlighting the role of RXR in normal or abnormal physiological processes. Convergence of 9cRA and PPAR signaling pathways through PPAR/RXRα heterodimerization is well established in mammals^54^, a crosstalk that is recently starting to be explored in gastropod mollusks affected by imposex. Two PPAR homologues have been already identified in a phylogenetically distant gastropod *Biomphalaria glabrata*^55^, indicating that these receptors are conserved in this group. As a whole, these results allow us to assume that PPARγ would function as a heterodimerization partner of RXR in the penis of both *B. globulosus* and *T. geversianus* males, and that RXR is the one that triggers imposex onset. However, many aspects of the RXR/PPAR system still remain to be explored.

Our results must be taken into account when performing environmental monitoring studies using imposex as a biomarker, since the studies of morphological structures (i.e. penis or vas deference) could hide physiological responses to marine pollutants. In the case of *T. geversianus*, this is the only muricid species described that is less sensitive to TBT contamination, developing secondary sexual characters only at high TBT concentrations^41^.

RXR was previously reported to be expressed at the mRNA level in penis from the marine muricid gastropods *T. clavigera*^56^, *N. lapillus*^21^ and *P. pansa*^30^, and at the protein level in *T. clavigera*^56,57^. However, to our knowledge, there is no information about PPARγ protein expression in marine gastropods. RXR and PPARγ interact in heterodimeric complexes after their activation by TBT^58^ or 9cRA, the biologically active metabolite of vitamin A^59^. It has been hypothesized that signaling through both proteins has a key role in male and female seasonal reproductive development^29,60^ and imposex, as previously mentioned. Whereas there is scant information regarding the physiological roles of RA in invertebrates, it is well known that signaling pathways through RA receptors exert a key role in embryo patterning and organogenesis in vertebrates^61^. 9cRA, is the natural ligand for mammalian RXRs, while the natural ligand of gastropod RXRs is currently unknown^62^. Vitamin A can either enter in a cascade producing retinal and RA, or undergo esterification to promote retinoid storage^63^. RA isomers were detected in testis, ovary and CNS of the caenogastropods *N. lapillus* and *Nassarius reticulatus*. However, they seem to be unable to store RA^27,64^. Finally, injection of TBT or 9cRA, into *N. lapillus* or *H. trunculus* males induces the outgrowth of reproductive structures^14,15^. All these data support the close relationship between retinoids and RXR/PPARγ in the development of male reproductive organs in marine gastropod mollusks.

The abnormal modulation of RXR signaling pathway by organotins seems to be the most accepted explanation of imposex development based on imposex induction assays together with the *in vitro* transcriptional activity of RXR^20,31^. Our findings, regarding the expression of RXR, contribute to the general understanding of the endocrine system in gastropods, and allow us to hypothesize that the degree of expression of RXR has direct implications on the sensitivity to imposex development.

We have also shown that imposex-affected female of *B. globulosus* collected from the polluted area (LPBH) of Nuevo Gulf, express RXR and PPARγ. These results are consistent with those obtained in species such as *T. clavigera*^56,65^, *N. lapillus*^66^, *H. trunculus*^15^, *P. pansa*^30^. However, our study is the first description of PPARγ protein expression in caenogastropod imposex-affected females. Regarding PPARγ actions, Pascoal *et al*.^67^ showed that the PPARγ ligand Rosi elicited the same imposex response in *N. lapillus* as TBT. However, Giraud-Billoud *et al*.^68^ reported that Rosi was not able to induce imposex in a phylogenetically distant gastropod *Pomacea canaliculata*. This difference indicates that different molecular mechanisms may regulate imposex induction in ampullariid and neogastropod species suggesting that the direct role of PPARγ in imposex deserves further investigation.

Males of *B. globulosus* that inhabit LPBH area are exposed to TBT^41,44^, PAHs^46^, trace metals and products from fishing industries^45,69^. These populations have increased oxidative stress responses compared to populations in CA beach^69^, indicating the negative effects of pollutants present at the harbour site on its physiological state. Our previous studies demonstrate the presence of high butyltins (TBT, DBT and MBT) levels in LPBH, both in sediments and in edible gastropods^41,44^. The sediments from LPBH in 2015^44^ exceeded the TBT limit concentration established by international organizations, this probably continues up to the present time, as occurs in other countries of Latin America^10–12^. This may explain our findings of imposex-affected females in the area. It has been shown using *in vitro* assays^70^, that PAHs enhance the effect of natural ligands of retinoid signaling pathway, indicating that these environmental pollutants may influence the differentiation process and the embryonic development mediated by retinoids.

Differences in RXR expression between males from LPBH and CA could be related to the pollutants present in the harbor area. However, this should be tested in controlled experiments exposing normal individuals to TBT, PAHs or trace metals separately. The regulation of RXR expression by TBT seems not to be conclusive. Domínguez-Ojeda and colleagues, reported a down regulation of RXR induced by TBT in different tissues of *P. pansa*, while no changes were observed in penis of males^30^. Similar results were obtained in *N. lapillus*, *H. trunculus* and *T. clavigera* where no changes in RXR mRNA expression were observed using penis of males after TBT treatment^15,66,71^. The ideal correlation between mRNA-protein may be affected by highly dynamic phases, such as cellular differentiation or stress response^72^. In our case, despite not being able to directly associate RXR protein expression to any specific factor, we found a difference in RXR protein between both sites, but not for PPARγ, probably indicating a major role of RXR in the induction of the imposex phenomenon in these species and probably other around the world.

Overall, our results clearly show that differences in RXR male penises expression between *B. globulosus* and *T. geversianus* do not affect the normal development of secondary sexual organs. Future cloning and functional studies with *B. globulosus* and *T. geversianus* RXRs might reveal its real contribution to the observed differences in imposex development and penis formation between both species.

## Supplementary information

Supplementary information.

## References

[1] Matthiessen, P. In *Endocrine Disrupters* 373–384 (John Wiley & Sons, Inc., 2013).

[2] Gibbs, P. E. & Bryan, G. W. In *Oceans ’87*. (ed IEEE) 1482–1487 (IEEE).

[3] Smith BS (1971). Sexuality in the american mud snail, Nassarius obsoletus say. J. Mollus Stud..

[4] Bryan GW, Gibbs PE, Hummerstone LG, Burt GR (1986). The decline of the gastropod Nucella lapillus around South-West England: Evidence for the effect of Tributyltin from antifouling paints. J. Mar. Biol. Assoc. U K..

[5] Goldberg ED (1986). TBT: An Environmental Dilemma. Environment: Sci. Policy Sustain. Dev..

[6] Laranjeiro F, Sánchez-Marín P, Oliveira IB, Galante-Oliveira S, Barroso C (2018). Fifteen years of imposex and tributyltin pollution monitoring along the Portuguese coast. Environ. Pollut..

[7] International Maritime Organization. & International Maritime Organization. Marine Environment Protection Committee. *Anti-fouling systems: International Convention on the Control of Harmful Anti-fouling Systems on Ships, 2001 (AFS 2001) and Guidelines for survey and certification of anti-fouling systems on ships (resolution MEPC.102(48))*. (International Maritime Organization, 2003).

[8] Schøyen M (2019). Levels and trends of tributyltin (TBT) and imposex in dogwhelk (Nucella lapillus) along the Norwegian coastline from 1991 to 2017. Mar. Environ. Res..

[9] Lahbib Y, Abidli S, Trigui-El Menif N (2018). First assessment of the effectiveness of the international convention on the control of harmful anti-fouling systems on ships in Tunisia using imposex in Hexaplex trunculus as biomarker. Mar. Pollut. Bull..

[10] Batista RM, Castro IB, Fillmann G (2016). Imposex and butyltin contamination still evident in Chile after TBT global ban. Sci. Total. Environ..

[11] Maciel DC (2018). Assessment of organotins and imposex in two estuaries of the northeastern Brazilian coast. Mar. Pollut. Bull..

[12] Castro ÍB, Iannacone J, Santos S, Fillmann G (2018). TBT is still a matter of concern in Peru. Chemosphere.

[13] van Gessellen N, Bouwman H, Averbuj A (2018). Imposex assessment and tributyltin levels in sediments along the Atlantic coast of South Africa. Mar. Environ. Res..

[14] Castro LFC (2007). Imposex induction is mediated through the Retinoid X Receptor signalling pathway in the neogastropod Nucella lapillus. Aquat. Toxicol..

[15] Abidli S (2013). Imposex development in Hexaplex trunculus (Gastropoda: Caenogastropoda) involves changes in the transcription levels of the retinoid X receptor (RXR). Chemosphere.

[16] Bettin C, Oehlmann J, Stroben E (1996). TBT-induced imposex in marine neogastropods is mediated by an increasing androgen level. Helgol. Mar. Res..

[17] Gibbs PE, Bryan GW, Pascoe PL (1991). TBT-induced imposex in the dogwhelk, Nucella lapillus: Geographical uniformity of the response and effects. Mar. Env. Res..

[18] Nishikawa J (2004). Involvement of the retinoid x receptor in the development of imposex caused by Organotins in gastropods. Env. Sci. Technol..

[19] Rossato M, Costa MB, de Castro ÍB, Pinho GLL (2018). Size, season and origin of gastropods matter in imposex assessments. Ecotoxicol. Environ. Saf..

[20] Horiguchi, T. Mode of Action of Organotins to Induce the Development of Imposex in Gastropods, *Focusing on Steroid and the Retinoid X Receptor Activation Hypotheses* 199–219, 10.1007/978-4-431-56451-5_9 (2017).

[21] Lima D (2011). Tributyltin-induced imposex in marine gastropods involves tissue-specific modulation of the retinoid X receptor. Aquat. Toxicol..

[22] Nishikawa J (2006). Imposex in marine gastropods may be caused by binding of organotins to retinoid X receptor. Mar. Biol..

[23] Urushitani H (2011). Cloning and characterization of retinoid X receptor (RXR) isoforms in the rock shell, Thais clavigera. Aquat. Toxicol..

[24] Helsen C (2012). Structural basis for nuclear hormone receptor DNA binding. Mol. Cell Endocrinol..

[25] Das BC (2014). Retinoic acid signaling pathways in development and diseases. Bioorg Med. Chem..

[26] Osz, J. *et al*. Structural basis of natural promoter recognition by the retinoid X nuclear receptor. *Scientific Reports* **5**, 1–10, 10.1038/srep08216, https://www.nature.com/articles/srep08216#supplementary-information (2015). 10.1038/srep08216 PMC4314640 25645674

[27] Gesto M, Castro LF, Santos MM (2013). Differences in retinoid levels and metabolism among gastropod lineages: imposex-susceptible gastropods lack the ability to store retinoids in the form of retinyl esters. Aquat. Toxicol..

[28] Gesto M (2016). Retinoid level dynamics during gonad recycling in the limpet Patella vulgata. Gen. Comp. Endocrinol..

[29] Sternberg RM, Hotchkiss AK, LeBlanc GA (2008). Synchronized expression of retinoid X receptor mRNA with reproductive tract recrudescence in an imposex-susceptible mollusc. Env. Sci. Technol..

[30] Domínguez-Ojeda D, Rojas-García EA, Robledo-Marenco ML, Barrón-Vivanco BS, Medina-Díaz IM (2014). Exposure to tributyltin chloride induces penis and vas deferens development and increases RXR expression in females of the purple snail (Plicopurpura pansa) Invertebrate Survival. Journal.

[31] Urushitani H (2018). Characterization and comparison of transcriptional activities of the retinoid X receptors by various organotin compounds in three prosobranch gastropods; Thais clavigera, Nucella lapillus and Babylonia japonica. Aquat. Toxicol..

[32] Urushitani H (2011). Cloning and characterization of retinoid X receptor (RXR) isoforms in the rock shell, Thais clavigera. Aquat. Toxicol..

[33] le Maire A, Bourguet W, Balaguer P (2010). A structural view of nuclear hormone receptor: endocrine disruptor interactions. Cell Mol. Life Sci..

[34] Horiguchi T (2008). Exposure to 9- retinoic acid induces penis and vas deferens development in the female rock shell, Thais clavigera. Cell Biol. Toxicol..

[35] Pascoal S (2013). Transcriptomics and *in vivo* tests reveal novel mechanisms underlying endocrine disruption in an ecological sentinel, Nucella lapillus. Mol. Ecol..

[36] Pastorino G (1993). The taxonomic status of Buccinanops d’Orbigny, 1841 (Gastropoda: Nassariidae). Veliger.

[37] Averbuj A, Penchaszadeh PE (2010). On the reproductive biology and impact of imposex in a population of Buccinanops monilifer from Mar del Plata, Argentina. J. Mar. Biol. Assoc. U K..

[38] Penchaszadeh PE, Averbuj A, Cledón M (2001). Imposex in gastropods from Argentina (South-Western Atlantic). Mar. Pollut. Bull..

[39] Griffin M, Pastorino G (2005). The genus Trophon Monfort, 1810 (Gastropoda: Muricidae) in the Tertiary of Patagonia. J. Paleontol..

[40] Pastorino G (2005). A revision of the genus Trophon Montfort, 1810 (Gastropoda: Muricidae) from southern South America. Nautilus.

[41] Bigatti G (2009). Biomonitoring of TBT contamination and imposex incidence along 4700 km of Argentinean shoreline (SW Atlantic: from 38S to 54S). Mar. Pollut. Bull..

[42] Primost MA, Bigatti G, Márquez F (2016). Shell shape as indicator of pollution in marine gastropods affected by imposex. Mar. Freshw. Res..

[43] APPM. *Administración Portuaria de Puerto Madryn. Available at*, http://www.appm.com.ar/estadisticas, (2017).

[44] Del Brio F (2016). Distribution and bioaccumulation of butyltins in the edible gastropod Odontocymbiola magellanica. Mar. Biol. Res..

[45] Primost MA, Gil MN, Bigatti G (2017). High bioaccumulation of cadmium and other metals in Patagonian edible gastropods. Mar. Biol. Res..

[46] Primost MA, Commendatore M, Torres PJ, Bigatti G (2018). PAHs contamination in edible gastropods from north Patagonian harbor areas. Mar. Pollut. Bull..

[47] Commendatore MG, Esteves JL (2007). An Assessment of Oil Pollution in the Coastal Zone of Patagonia, Argentina. Environ. Manag..

[48] Commendatore, M. G. *et al*. BTs, PAHs, OCPs and PCBs in sediments and bivalve mollusks in a mid-latitude environment from the Patagonian coastal zone. *Environmental toxicology and chemistry/SETAC*, 10.1002/etc.3134 (2015). 10.1002/etc.3134 26118658

[49] Gibbs, P. E. & Bryan, G. W. Biomonitoring of Tributyltin (TBT) Pollution using the Imposex Response of Neogastropod Molluscs. Biomonitoring of Coastal Waters and Estuaries. *Biomonitoring of Coastal Waters and Estuaries*, 205–226 (1994).

[50] Lowry OH, Rosebrough NJ, Farr AL, Randall RJ (1951). Protein measurement with the Folin phenol reagent. J. Biol. Chem..

[51] Schneider CA, Rasband WS, Eliceiri KW (2012). NIH Image to ImageJ: 25 years of image analysis. Nat. Meth.

[52] Castro LF (2007). Imposex induction is mediated through the Retinoid X Receptor signalling pathway in the neogastropod Nucella lapillus. Aquat. Toxicol..

[53] Sternberg RM, Hotchkiss AK, LeBlanc GA (2008). Synchronized expression of retinoid X receptor mRNA with reproductive tract recrudescence in an imposexsusceptible mollusc. Environ. Sci. Technol..

[54] Kliewer SA, Umesono K, Noonan DJ, Heyman RA, Evans RM (1992). Convergence of 9-cis retinoic acid and peroxisome proliferator signalling pathways through heterodimer formation of their receptors. Nature.

[55] Fong PP (2015). The Nuclear Receptors of Biomphalaria glabrata and Lottia gigantea: Implications for Developing New Model Organisms. PLoS One.

[56] Horiguchi T, Nishikawa T, Ohta Y, Shiraishi H, Morita M (2007). Retinoid X receptor gene expression and protein content in tissues of the rock shell Thais clavigera. Aquat. Toxicol..

[57] Horiguchi T, Urushitani H, Ohta Y, Iguchi T, Shiraishi H (2010). Establishment of a polyclonal antibody against the retinoid X receptor of the rock shell Thais clavigera and its application to rock shell tissues for imposex research. Ecotoxicology.

[58] le Maire A (2009). Activation of RXR-PPAR heterodimers by organotin environmental endocrine disruptors. EMBO Rep..

[59] Hiromori Y, Aoki A, Nishikawa J, Nagase H, Nakanishi T (2015). Transactivation of the human retinoid X receptor by organotins: use of site-directed mutagenesis to identify critical amino acid residues for organotin-induced transactivation. Metallomics: Integr. biometal Sci..

[60] Sternberg RM, Gooding MP, Hotchkiss AK, LeBlanc GA (2010). Environmental-endocrine control of reproductive maturation in gastropods: implications for the mechanism of tributyltininduced imposex in prosobranchs. Ecotoxicology.

[61] Cunningham TJ, Duester G (2015). Mechanisms of retinoic acid signalling and its roles in organ and limb development. Nat. Rev. Mol. Cell Biol..

[62] Horiguchi, T. In *Biological Effects by Organotins* (ed Toshihiro Horiguchi) 199-219 (Springer Japan, 2017).

[63] Theodosiou M, Laudet V, Schubert M (2010). From carrot to clinic: an overview of the retinoic acid signaling pathway. Cell Mol. Life Sci..

[64] André A, Ruivo R, Gesto M, Castro LFC, Santos MM (2014). Retinoid metabolism in invertebrates: When evolution meets endocrine disruption. Gen. Comp. Endocrinol..

[65] Ip JCH, Leung PTY, Ho KKY, Qiu JW, Leung KMY (2016). *De novo* transcriptome assembly of the marine gastropod Reishia clavigera for supporting toxic mechanism studies. Aquat. Toxicol..

[66] Lima D (2011). Tributyltin-induced imposex in marine gastropods involves tissue-specific modulation of the retinoid X receptor. Aquat. Toxicol..

[67] Pascoal S (2013). Transcriptomics and *in vivo* tests reveal novel mechanisms underlying endocrine disruption in an ecological sentinel, Nucella lapillus. Mol. Ecol..

[68] Giraud-Billoud M, Castro-Vazquez A (2019). Aging and retinoid X receptor agonists on masculinization of female Pomacea canaliculata, with a critical appraisal of imposex evaluation in the Ampullariidae. Ecotoxicol. Environ. Saf..

[69] Primost MA, Sabatini SE, Di Salvatore P, Ríos De Molina MC, Bigatti G (2015). Oxidative stress indicators in populations of the gastropod Buccinanops globulosus affected by imposex. J. Mar. Biol. Assoc. U K..

[70] Beníšek M, Bláha L, Hilscherová K (2008). Interference of PAHs and their N-heterocyclic analogs with signaling of retinoids *in vitro*. Toxicol. Vitro.

[71] Horiguchi T, Nishikawa T, Ohta Y, Shiraishi H, Morita M (2010). Time course of expression of the retinoid X receptor gene and induction of imposex in the rock shell, Thais clavigera, exposed to triphenyltin chloride. Anal. Bioanal. Chem..

[72] Liu Y, Beyer A, Aebersold R (2016). On the Dependency of Cellular Protein Levels on mRNA Abundance. Cell.

