# Supplementary material for: RXR Expression in Marine Gastropods with Different Sensitivity to Imposex Development
Source: Sci Rep. 2020 Jun 11;10:9507. doi: 10.1038/s41598-020-66402-1 (PMC7289818; doi:10.1038/s41598-020-66402-1)

RXR EXPRESSION IN MARINE GASTROPODS WITH DIFFERENT SENSITIVITY TO IMPOSEX DEVELOPMENT

Sebastián Giulianelli[1,2], Mónica A. Primost[1,3], Claudia Lanari[2] and Gregorio Bigatti[1,4,5]

[1]Instituto de Biología de Organismos Marinos. IBIOMAR-CCT CENPAT-CONICET. Puerto Madryn, Argentina.

[2]Instituto de Biología y Medicina Experimental. IBYME-CONICET. Buenos Aires, Argentina.

[3]Grupo de Investigación y Desarrollo Tecnológico en Acuicultura y Pesca (GIDTAP). Universidad Tecnológica Nacional. Facultad Regional Chubut (UTN-FRCh), Argentina.

[4]Universidad Nacional de la Patagonia San Juan Bosco. Puerto Madryn, Argentina.

[5]Universidad Espíritu Santo, Ecuador.

Supplementary Figure S1:

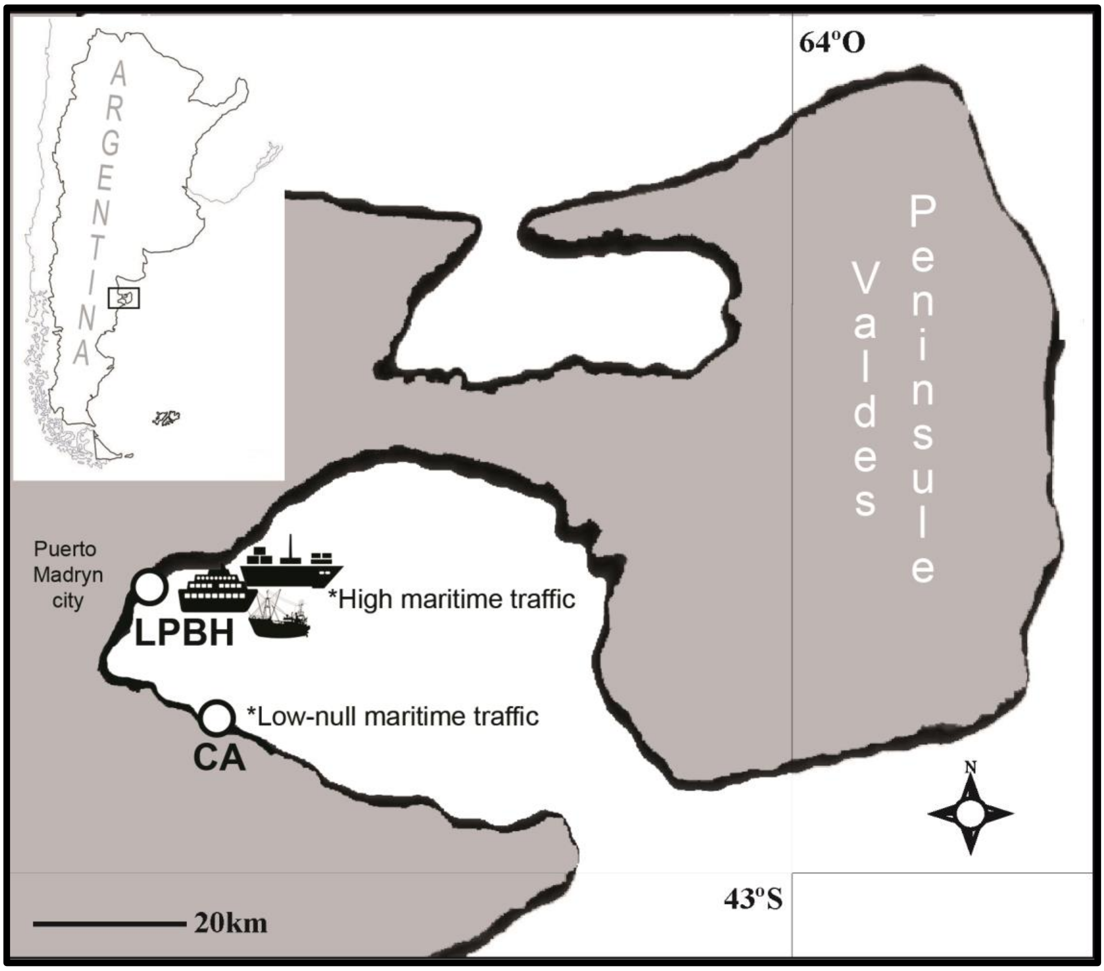

Figure S1. Study area and location of the sampled sites. The map was sourced from Google Earth Pro (v7.3) and modified in Adobe Illustrator (v16.0.0). CA: Cerro Avanzado beach, LPBH: Luis Piedra Buena Harbor.

Supplementary Figure S2:

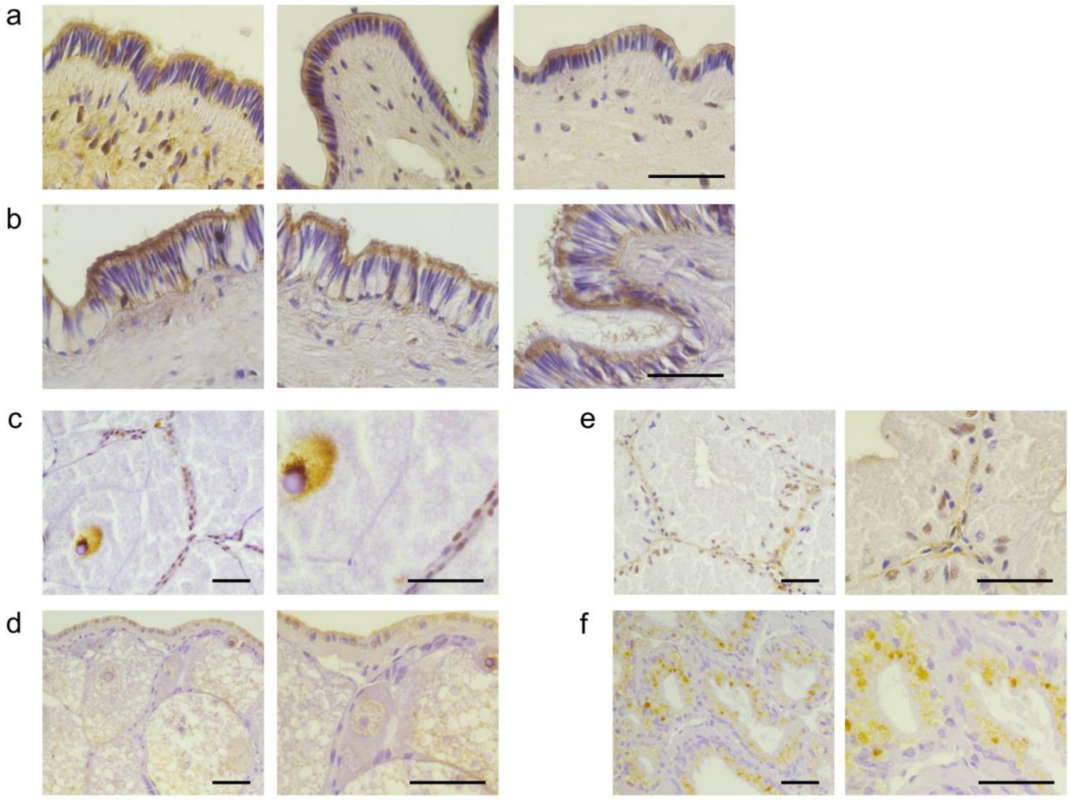

Figure S2. Immunohistochemical staining for RXR. (a and b) Epidermal region of male penis from *B. globulosus* and *T. geversianus*, respectively. Bar: 50 µm. (c and d) Gonads of *B. globulosus* and *T. geversianus* females, respectively. Bar: 50 µm. (e and f) Digestive glands of *B. globulosus* and *T. geversianus* females, respectively. Bar: 50 µm.

Supplementary Figure S3: Full-length gels from Figure 2

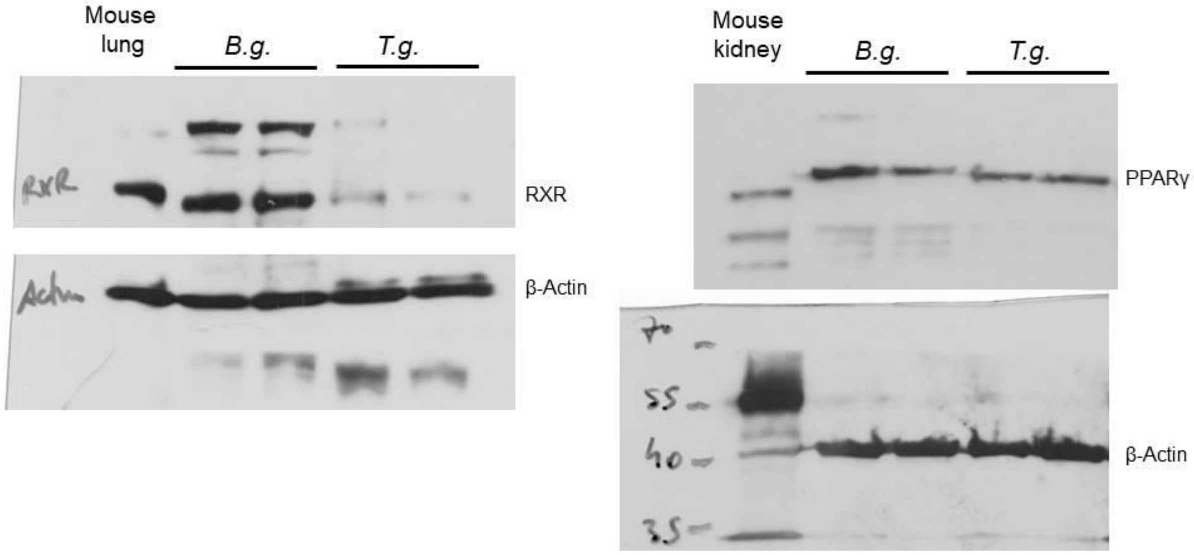

Supplementary Figure S4: Full-length gels from Figure 3

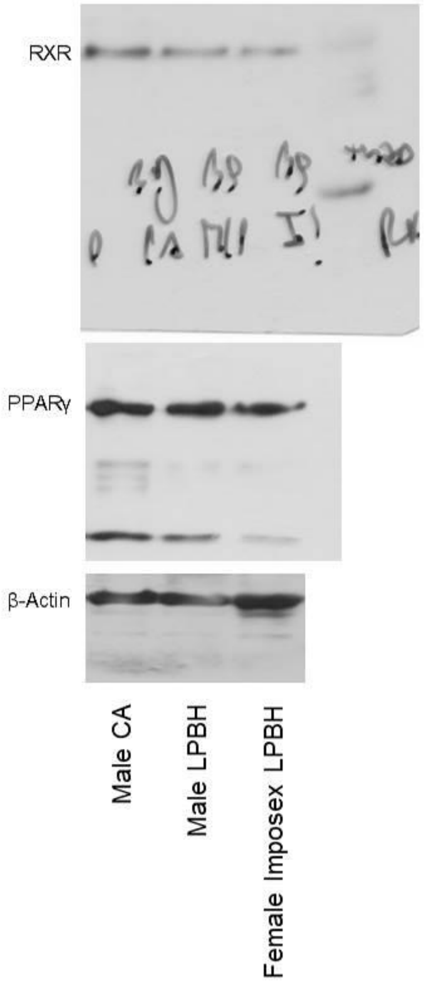

Supplement: Supplementary file 1 — Supplementary information. [file 41598_2020_66402_MOESM1_ESM.pdf]
